# Capillary leak syndrome and aseptic meningitis in a patient with Kawasaki disease: A case report

**DOI:** 10.1097/MD.0000000000010716

**Published:** 2018-06-18

**Authors:** Yufeng Zhang, Han Wan, Maosheng Du, Huiling Deng, Jia Fu, Yu Zhang, Xiaoyan Wang, Ruiqing Liu

**Affiliations:** a Second Department of Infectious Diseases, Xi’an Children's Hospital; b Department of Hepatobiliary Surgery, The 521 Hospital of China Ordnance Industry; c Outpatient Office, Xi’an Children's Hospital, Xi’an, Shaanxi Province, China.

**Keywords:** aseptic meningitis, capillary leak syndrome, Kawasaki disease

## Abstract

**Rationale:**

Kawasaki disease (KD) is an acute vasculitis of childhood, coronary complications are the most serious and classic complications of this disease. However, simultaneous complications such as systemic capillary leak syndrome (CLS) and aseptic meningitis are rarely reported.

**Patient concerns:**

A 19-month-old boy had continuous fever for 6 days, rash for 3 days, and somnolence for 1 day.

**Diagnoses:**

The boy was diagnosed with KD presenting with SCLS and aseptic meningitis.

**Interventions:**

He was treated with gamma globulin (2 g/kg) for 1 day, mannitol and furosemide to reduce intracranial pressure, human albumin to correct hypoproteinemia, methylprednisolone to control inflammation, and both aspirin and dipyridamole for anticoagulation.

**Outcomes:**

After treatment, the patient recovered well. At one year follow-up, the patient was asymptomatic and showed no recurrence of skin rash.

**Lessons:**

The incidence of KD has recently increased and cardiovascular complications are frequently reported. This may be combined with systemic damage, however, the combination of SCLS and aseptic meningitis is rarely reported, therefor, children who have SCLS, aseptic meningitis and unexplained fever >5 days, KD should be taken into account. Early diagnosis and timely treatment can reduce complications induced by KD.

## 1 Introduction

Kawasaki disease (KD) is a multisystem and self-limited vasculitis syndrome with an unknown etiology. It was first reported by Kawasaki.^[1]^ There is no specific diagnostic test for KD to date, and diagnosis is based on clinical signs and symptoms.^[2]^ Coronary complications are the most serious and classic complications of this disease. Simultaneous complications such as systemic capillary leak syndrome (SCLS) and aseptic meningitis are rarely reported. Here, we describe a case of KD presenting with SCLS and aseptic meningitis in a 19-month-old boy.

## 2 Case report

A previously healthy 19-month-old boy was admitted to our hospital because of continuous fever for 6 days, rash for 3 days, and somnolence for 1 day. One day before admission, the patient developed red, cracked lips, red soles and palms, neck stiffness, facial and limb edema, accompanied by somnolence and vomiting 4 times a day. Before admission, he was prescribed cefmenoxime for suspected respiratory tract infection, but the patient's condition worsened. His family history was not significant.

A physical examination on admission revealed normal blood pressure, pulse rate was 128 beats/minute with a regular rhythm, and his body temperature was 37.9^o^C. Heart sounds did not reveal any abnormality, respiratory rate was slightly higher than normal (31 breaths/minute), and pulmonary auscultation was normal. The face and limbs showed mild nonpitting edema. Bilateral cervical lymph nodes were enlarged (>1.5 cm in diameter). Nonexudative conjunctivitis, red lips, a strawberry tongue, red oral mucosa, scarlet fever-like rash, and a stiff neck were observed. Physical examination of the nervous system showed typical signs of meningeal irritation, including neck rigidity, Kernig's sign, and Brudzinski's sign. No other abnormalities were found.

On admission, laboratory examination showed hemoglobin of 10^4^ g/L, leukocytosis of 23.12×10^9^/L (79.5% neutrophils; 15.1% lymphocytes), platelet count of 168×10^9^/L, C-reactive protein of 163 mg/dL, and erythrocyte sedimentation rate of 67 mm/1st h. Other blood biochemical tests including glucose, triglycerides, calcium, and procalcitonin were all within reference limits. Total levels of IgG, IgA, and IgM in blood were normal. Liver function tests showed that total protein (TP) and albumin (ALB) were decreased (TP: 49.5 g/L; ALB: 24.9 g/L; reference range: 57–80 g/L; 37–51 g/L), total bilirubin (TBIL), and direct bilirubin (D-BIL) were slightly increased (TBIL: 31.2 μmol/L; D-BIL: 19.2 μmol/L; reference range: 3.4–20.5 μmol/L; 0–6.8 μmol/L), alanine aminotransferase (ALT), and aspartate aminotransferase (AST) were slightly elevated (ALT: 78 IU/L; AST: 52 IU/L; reference range: 10–35 IU/L), and total bile acid (125 μmol/L; reference range: 0–10 μmol/L) was also increased. Myocardial enzyme spectrum, renal function, electrolytes, blood coagulation, and blood ammonia were normal. Urinary calculus and urine protein were (−). Serological tests for various infectious agents including Epstein–Barr virus, varicella zoster virus, cytomegalovirus, HIV I and II, hepatitis A, B and C viruses, enterovirus, syncytial virus, flu virus A and B, parainfluenza 1, 2, and 3, rubella virus, herpes simplex virus, *Mycoplasma pneumoniae*, *Chlamydia pneumonia,* and blood culture were all negative. Tumor markers were also negative.

Other relevant tests and examinations during hospitalization were performed and the results were as follows: pressure was mildly elevated (26 cm H_2_O) in examination of cerebrospinal fluid (CSF), protein was 494 mg/L, glucose, chloride and cell numbers were normal, and Gram stain and Indian ink examination for *Cryptococcal neoformans* capsule and CSF cultures were negative. MRI of the brain, chest roentgenogram, electrocardiogram and electroencephalogram were normal. Ultrasonography of the abdomen showed that the gallbladder volume was increased, the gallbladder wall was thickened, there was no obvious dilatation of the bile duct, and the liver and spleen were normal. Abdominal computed tomography (CT) revealed a significant increase in gallbladder volume and ascites. Chest CT showed bilateral lower lobe exudative lesions with bilateral pleural effusion. Color sonography showed bilateral coronary artery dilation (with a left coronary artery diameter of 2.9 mm and a right coronary artery diameter of 2.8 mm), left ventricular function was normal, and there was a small amount of pericardial effusion.

Following admission, KD was diagnosed and was found to be complicated by capillary leak syndrome and aseptic meningitis. The boy was treated with gamma globulin (2 g/kg) for 1 day, mannitol and furosemide to reduce intracranial pressure, human albumin to correct hypoproteinemia, methylprednisolone to control inflammation, and both aspirin and dipyridamole for anticoagulation.

The boy's symptoms improved after 3 days, his body temperature returned to normal, edema gradually improved, his spirit improved, and peeling of the digits appeared. On the day of discharge, ultrasonography was performed and no effusion was found in the serous cavities, liver and gallbladder were normal, blood leucocyte count was 10.1 × 10^9^/L, platelet count was 722 × 10^9^/L, liver function and C-reactive protein were normal, and erythrocyte sedimentation rate was 50 mm/1st h. After discharge, the patient continued taking oral aspirin and dipyridamole.

One month later, the findings on color sonography were normal and all laboratory values were within normal ranges. At 1 year follow-up, the patient was asymptomatic and showed no recurrence of skin rash.

## 3 Discussion

KD is an acute vasculitis of childhood. Its clinical presentation is well known, and coronary artery aneurysms are its classic complications. SCLS and aseptic meningitis are rare presentations of the disease. Here, we present a case of KD associated with simultaneous SCLS and aseptic meningitis. The diagnosis of KD in this case was based on the following evidence: fever for more than 5 days after ineffective antibiotic treatment, enlarged lymph nodes, nonexudative conjunctivitis, red lips, a strawberry tongue, red oral mucosa, scarlet fever-like rash, peeling fingers and toes, coronary artery dilation on color sonography, elevated C-reactive protein, and platelets. All viral antibody testing and bacterial culture results were negative.

Systemic capillary leak syndrome is a rare but devastating disease which was first described in 1960.^[3]^ It is characterized by a series of clinical symptoms, which are caused by multiple factors, such as immune-mediated extensive capillary endothelial damage and capillary endothelium hyperpermeability leading to extravascular leakage of plasma into the interstitial area.^[4]^ The main manifestations are mucocutaneous and visceral edema (not originating from the heart, kidney and liver), multiple serous cavity effusions, hypoalbuminemia, hemoconcentration, and decreased central venous pressure. Severe cases may develop pulmonary interstitial exudation, hypovolemic shock and hypoperfusion-induced organ dysfunction. The disease can be idiopathic (Clarkson syndrome) ^[5]^ or secondary to other diseases and treatments.^[6]^ However, CLS in children often has a clear etiology, particularly severe infection or trauma, extracorporeal circulation and mechanical ventilation, or drug poisoning,^[7]^ but CLS secondary to KD is rare and there are a few reports in the literature.^[8]^

Diagnosis of CLS is mainly based on clinical manifestations and lacks specific prevention and treatment options. The principle of treatment is to control its progression, maintain organ function, maintain effective blood volume, and improve capillary permeability.^[9]^ For liquid expansion, hydroxyethyl starch is preferred as it can improve endothelial function, reduce inflammation, effectively promote the flow of liquids and thus is often used in the clinic. Glucocorticoids can improve capillary permeability, which can be used to suppress inflammation, and are combined with diuretics to reduce volume load. During the disease course in our patient, there were multiple serous cavity effusions and soft tissue edema, and as cardiac factors were excluded, these suggested that fluid was oozing from blood vessels. However, there was no reduction in blood volume, and the decrease in plasma albumin could not be explained by decreased synthesis in the liver or excessive discharge by the kidneys; therefore, the clinical diagnosis of CLS was made. CLS in this case was not serious as no hypoperfusion, pulmonary interstitial edema or liquid reflux without circulatory congestion were observed, and the patient recovered quickly, which may have been attributed to the timely control of KD.

KD with neurologic complications is uncommon. Aseptic meningitis often occurs in the acute stage, and manifested mainly as increased intracranial pressure, resulting in symptoms such as headache, vomiting, anterior fontanelle bulge, and meningeal irritation, and seizures can occur in some cases. On routine inspection of CSF, increased white blood cells, particularly lymphocytes are observed. Sugar and chloride are usually normal, and protein can be slightly increased and may be accompanied by electroencephalogram abnormalities. If the above clinical manifestations are present, the diagnosis of viral meningitis can be established. Therefore, in children with the diagnosis of viral meningitis who have long-term fever, it is necessary to determine whether bacterial meningitis is present and attention should be paid to the possibility of KD with aseptic meningitis. The diagnosis of KD with aseptic meningitis in our patient was made on the basis of clinical symptoms including fever, drowsiness and signs of meningeal irritation. Cerebrospinal fluid examination showed elevated CSF protein, glucose, and chloride with a normal number of cells. CSF culture showed no bacterial growth, and all viral antibody testing results were negative, excluding purulent meningitis, tuberculous meningitis and cryptococcal meningitis, hence a final diagnosis of KD with aseptic meningitis. The addition of mannitol to reduce intracranial pressure is important in the treatment of KD with aseptic meningitis, as the clinical symptoms can be resolved after the recovery of intracranial pressure after dehydration. Our patient recovered well without neurologic sequelae. KD with aseptic meningitis is a noninfectious meningitis, and the pathogenesis has not been fully elucidated. Possible mechanisms may involve systemic vasculitis due to the inflammatory response in pial vessels, however, this speculation remains to be confirmed by further studies.

## 4 Conclusions

The incidence of KD has recently increased and cardiovascular complications are frequently reported. This may be combined with systemic damage, however, the combination of SCLS and aseptic meningitis is rarely reported, therefor, children who have SCLS, aseptic meningitis and unexplained fever >5 days, KD should be taken into account. Early diagnosis and timely treatment can reduce complications induced by KD.

## Acknowledgments

We thank the doctors of the Department of Infectious Diseases of Xi’an Children's Hospital for their help in data collection.

## Author contributions

**Conceptualization:** Yufeng Zhang, Huiling Deng.

**Data curation:** Yufeng Zhang, Han Wan, Huiling Deng, Jia Fu, Yu Zhang.

**Formal analysis:** Yufeng Zhang, Han Wan, Maosheng Du, Yu Zhang.

**Funding acquisition:** Huiling Deng.

**Investigation:** Maosheng Du, Huiling Deng, Xiaoyan Wang.

**Project administration:** Ruiqing Liu.

**Resources:** Jia Fu.

**Writing – original draft:** Huiling Deng.
